# Translocator Protein-18 kDa (TSPO) Positron Emission Tomography (PET) Imaging and Its Clinical Impact in Neurodegenerative Diseases

**DOI:** 10.3390/ijms18040785

**Published:** 2017-04-07

**Authors:** Anne-Claire Dupont, Bérenger Largeau, Maria Joao Santiago Ribeiro, Denis Guilloteau, Claire Tronel, Nicolas Arlicot

**Affiliations:** 1 CHRU Tours, 2 Boulevard Tonnellé, 37044 Tours, France; berenger.largeau@etu.univ-tours.fr (B.L.); maria.ribeiro@univ-tours.fr (M.J.S.R.); denis.guilloteau@univ-tours.fr (D.G.); nicolas.arlicot@univ-tours.fr (N.A.); 2 Institut National de la Santé et de la Recherche Médicale U930, 10 Boulevard Tonnellé, 37032 Tours, France; claire.tronel@univ-tours.fr

**Keywords:** microglial activation, neuroinflammation, PET, radiopharmaceutical, TSPO, neurodegenerative diseases, psychiatric disorders

## Abstract

In vivo exploration of activated microglia in neurodegenerative diseases is achievable by Positron Emission Tomography (PET) imaging, using dedicated radiopharmaceuticals targeting the translocator protein-18 kDa (TSPO). In this review, we emphasized the major advances made over the last 20 years, thanks to TSPO PET imaging, to define the pathophysiological implication of microglia activation and neuroinflammation in neurodegenerative diseases, including Parkinson’s disease, Huntington’s disease, dementia, amyotrophic lateral sclerosis, multiple sclerosis, and also in psychiatric disorders. The extent and upregulation of TSPO as a molecular biomarker of activated microglia in the human brain is now widely documented in these pathologies, but its significance, and especially its protective or deleterious action regarding the disease’s stage, remains under debate. Thus, we exposed new and plausible suggestions to enhance the contribution of TSPO PET imaging for biomedical research by exploring microglia’s role and interactions with other cells in brain parenchyma. Multiplex approaches, associating TSPO PET radiopharmaceuticals with other biomarkers (PET imaging of cellular metabolism, neurotransmission or abnormal protein aggregates, but also other imaging modalities, and peripheral cytokine levels measurement and/or metabolomics analysis) was considered. Finally, the actual clinical impact of TSPO PET imaging as a routine biomarker of neuroinflammation was put into perspective regarding the current development of diagnostic and therapeutic strategies for neurodegenerative diseases.

## 1. Microglia

Microglial cells are the resident macrophages of the central nervous system (CNS) that play different roles in both physiological and pathological conditions, by maintaining brain parenchyma integrity and involving in a wide range of neurodegenerative diseases. Microglia represent approximately 5–15% of all cells in the human brain [1,2]. The brain homeostasis is achieved in part though the ability of microglia to regulate inflammation, such as cytotoxicity, repair and regeneration [3]. In parallel to their well-known immune-modulatory functions, microglia are highly dynamic cells which contribute to the synaptic remodelling/plasticity as well as synaptogenesis, synaptic transmission and pruning [4].

Several cellular actors including neurons, astrocytes, T-cells and the blood brain barrier (BBB) modulate microglial function and form a very dynamic network [5]. In particular, microglia have been proposed to act as sensors, effectors and injury recipients of the brain homeostasis breakdown [6]. In fact, upon injury, various afferents stimuli (e.g., soluble factors, cellular interactions [7,8]) induced morphological (i.e., amoeboid, rod, multi-nucleated, epithelioid or dystrophic state) and functional changes of microglia, known as microglial “activation” [6,9].

In front of homeostatic disturbances (e.g., vascular or tissue damage), microglial cells shift from sensing activity to a reactive state. Originally based on peripheral monocytes/macrophages, the reactive phenotype of microglial cells is dichotomized into “classical activation” or M1, pro-inflammatory and “alternative activation” or M2, anti-inflammatory reactions. According to this concept, microglia phenotypes are defined by triggering responses to cytokines and microbial agents. In fact, interferon-γ from T helper cell type 1 (Th1) causes M1 activation, a response usually associated with the struggle against intracellular pathogens. In M1 state, macrophages release pro-inflammatory cytokines such as IL-1β, IL-6, TNF-α or ROS/RNS. On the other hand, the secretion of IL-4 or IL-13 from Th2 promotes M2a polarization and supports tissue repair/regeneration. M2b cells are induced by the immune complex, secrete IL-10 and regulate the immune response [10]. When macrophages release TGF-β or IL-10 (M2c reaction), anti-inflammatory effects are observed [7,11]. Microglia polarization/action is a process highly context-dependent [12] (nature of activating stimulus; e.g., PAMPS and/or DAMPS) which is integrated in a time-dependent fashion (acute versus chronic injury) [6]. 

## 2. TSPO as a Target of Activated Microglia PET Imaging

### 2.1. TSPO

First described as a peripheral benzodiazepine receptor (PBR), the translocator protein-18 kDa (TSPO) has been fortuitously identified by Braestrup et al., using rat kidneys as a control tissue during central benzodiazepine receptor (CBR) binding studies using [^3^H]-diazepam. This secondary binding site for diazepam, which is distinct in its structure from the CBR, was shown to be abundantly distributed in peripheral tissues [13]. It has been renamed as 18-kDa translocator protein (TSPO) to better reflect the cellular functions and tissue distribution of this protein [14]. TSPO is a highly hydrophobic five transmembrane domain protein mainly situated in the outer mitochondrial membrane. TSPO is widely distributed in most peripheral organs including kidneys, nasal epithelium, adrenal glands, lungs and heart, whilst the highest concentrations are in the steroid producing tissues; is also minimally expressed in resting microglial cells in the healthy brain [15]. Although modestly expressed in healthy brain parenchyma, TSPO is substantially upregulated predominantly in microglia cells in a number of neurodegenerative and neuroinflammatory diseases during the microglia activation process [16]. In addition to activated microglia, Lavisse et al. showed that reactive astrocytes could also overexpress TSPO using a model of selective astrocyte activation in the rat striatum [17]. Likewise, after brain injury induced by focal cerebral ischemia, both microglia and astrocytes have been found to overexpress TSPO [18], with a dissimilar distribution within the infarcted lesion. Thus, astrocytes were mainly observed in the rim surrounding the lesion core, whereas microglia were abundant in the core of infarction [19]. Furthermore, cells of the mononuclear phagocyte lineage, such as peripheral macrophages, also express the TSPO and, in the case of a disrupted BBB, also infiltrate the damaged CNS due to the vascular permeability increased by neuroinflammation [20].

TSPO is thought to be involved in a wide array of vital cellular functions including porphyrin transport and heme synthesis, regulation of cell proliferation, steroid biosynthesis and programmed cell death [21]. TSPO’s functions in metabolic homeostasis remain unclear, especially in the supra-physiologic stimulation of microglia. Most studies point out that high TSPO expression attenuates ROS generation [22,23]. Gatliff and Campanella [24] recently specify to what extent TSPO drives the mitochondrial quality control. Indeed, more than TSPO (over)expression, it seems to be the expression ratio of TSPO to VDAC1 (voltage-dependent anion channel 1) that regulates ROS production. Subsequently, this ROS generation leads to an accumulation of defective mitochondria due to an ineffective mitophagy PARK2-dependent mitochondriopathy [24], which is involved in several neurodegenerative diseases such as Parkinson’s disease (PD) [25], Huntington’s disease (HD) [26] and Alzheimer‘s disease (AD) [27]. Others authors suggest a role of TSPO in oxidative stress by directly facilitating the breakdown of porphyrin [28]. The use of TSPO ligands in rodent models of neuroinflammation (e.g., kainic acid excitoxicity [29] or intracerebroventricular infusion of lipopolysaccharide (LPS) [30]) highlighted TSPO’s involvement in hippocampal gliosis and especially in microgliosis.

The colocalization of TSPO upregulation and activated glia has been observed in human degenerative disorders including AD, PD, multiple sclerosis (MS), (HD) [31] and amyotrophic lateral sclerosis (ALS) [32]. As a result, this increased density of TSPO in CNS diseases has been considered to be a relevant and an early biomarker to evaluate neuroinflammation over disease progression [33].

Positron Emission Tomography (PET) imaging of microglia has widely grown over the last 25 years, through the development of radiopharmaceuticals targeting several molecular biomarkers of microglial activation. In this context, many biological targets of neuroinflammation for both diagnosis and therapeutic purposes have been identified. Among them, TSPO has become the reference molecular biomarker for microglia PET imaging. Thus, TSPO PET imaging has been used for both improving the knowledge regarding the role of neuroinflammation in CNS diseases and to assess the efficacy of novel anti-inflammatory therapeutic strategies. Yet, the upregulation of TSPO PET radioligands’ binding is not discriminant between activated microglia, astrocytes and infiltrated macrophages. Indeed, in AD, Venneti et al. [34] observed that astrocytes are not a negligible factor in the increased TSPO PET radioligand binding. Moreover, TSPO overexpression in both astrocytes and microglia seems to be temporally distinct depending on the stage of disease and the disease itself [35]. Thus, TSPO PET imaging is not selective of microglia activation, but depicts a broader multicellular inflammatory reaction. 

In this section, we summarize the progress being made in both the involvement of microglia in various neurologic conditions and the understanding of microglial activation in CNS disorders thanks to TSPO PET molecular imaging.

### 2.2. TSPO PET Tracers

A wide variety of endogenous compounds like diazepam, cholesterol or porphyrin interacts with TSPO at distinct binding sites. Because of the availability of high affinity and selective ligands, which can be labeled with various radioisotopes (^11^C, ^18^F), the distribution of TSPO can be visualized with PET technique. Historically, the benzodiazepine ^11^C-Ro5-4864 was the first ligand able to discriminate peripheral from central benzodiazepine receptors. Then, the most widely used TSPO PET radiopharmaceutical, namely ^11^C-(R)-PK11195, an isoquinoline carboxamide developed in the early 1980s, was the first non-benzodiazepine type compound identified to bind to TSPO with high affinity (human Kd = 2 nM) [36]. However, ^11^C-(R)-PK11195 clinical usefulness is narrowed by several major limitations, including the short half-life of carbon-11, a low brain bioavailability and a poor signal-to-noise ratio due to high nonspecific binding [37]. To counteract these drawbacks, there has been a great effort for the development of second generation TSPO PET radiotracers [38,39,40], including phenoxyarylacetamides derivatives labeled with carbon-11 (^11^C-PBR28, 11C-DAA1106) or fluorine-18 (^18^F-FEPPA, ^18^F-PBR06), imidazopyridines derivatives (11C-CLINME) and pyrazolopyrimidines derivatives (^18^F-DPA-714). Indeed, a single nucleotide polymorphism in the TSPO gene (rs6971) leading to an amino-acid substitution (A147T) has shown to be the cause of binding affinity properties variation with a huge heterogeneity in PET images and their associated quantitative data. The fact that the second-generation radiopharmaceuticals have been found to be sensitive to this polymorphism leads searchers to develop rs6971-insensitive radioligands. These rs6971-insensitive radioligands, called third generation TSPO tracers, include ^11^C-ER176 [41] and flutriciclamide (^18^F-GE180) [42]. Nonetheless, the clinical relevance of these compounds remains to be confirmed [41]. 

TSPO PET tracers have been investigated in various preclinical and clinical studies and we have now reported major advances provided by the development of new tracers (Table 1). 

## 3. Clinical Input of TSPO PET Imaging in Neurodegenerative Diseases

### 3.1. Parkinson’s Disease and Related Syndromes

Parkinson’s disease (PD) is a common age-related neurodegenerative disorder that mainly affects the motor system. Symptoms include resting tremor, asymmetrical bradykinesia and muscular rigidity. PD results from the progressive degeneration of dopaminergic neurons, initially in the substantia nigra, which causes a clinical parkinsonism when the loss of the striatal dopamine reaches to 60% [44,45,46]. PD is neuropathologically characterized by a neuronal rarefaction in the substantia nigra pars compacta and the presence of neuronal Lewy bodies (LB) inclusions, which are abnormal aggregates of α-synuclein [47]. Since the discovery of activated microglia in a post mortem study of PD brains, objectified by a large amount of HLA-DR-positive microglial cells in the locus niger, “neuroinflammation” became one of the main culprits in the pathogenesis and in the progression of PD [48]. Correlation analysis between TSPO tracer binding potential values and clinical parameters show that microglial activation PET imaging should predict the symptom severity, but only in early-stage PD patients (Table 2) [49,50,51]. Indeed, the nigral ^11^C-PK11195 accumulation was significantly positively correlated with the motor severity of the UPDRS assessment (unified Parkinson’s disease rating scale) in drug-naïve PD patients [49]. In addition, PD patients presented significantly increased ^11^C-PK11195 uptake in the basal ganglia (striatum and pallidum), thalamus, brainstem and frontal, temporal and cortical regions [51,52]. This TSPO overexpression in different cerebral regions supports the notion that microglial activation is not confined to nigrostriatal dopaminergic neurons within PD pathophysiology. Moreover, identified by positive CD68 immunoreactivity, amoeboid activated microglial cells were identified in both the striatum and the hippocampus of PD patients, suggesting that an inflammatory response occurs to the active α-synuclein pathology in the hippocampus area [53]. Furthermore, TSPO PET imaging shows that the microglial shift, from a sensing to a reactive state, takes place at the early stage of PD [49,54] and can occur even without dementia [52,55]. This was corroborated by reported data from a rat model of PD, where the activation of microglia seems to precede dopaminergic neuronal cell loss [56]. Given the fact that a chronological spread of LB inclusions occurred in α-synucleinopathies [57], and these α-synuclein can directly provoke microglial activation [58], we could expect to find, in a time-dependent fashion, a widespread TSPO signal in PET imaging. Interestingly, Gerhard and colleagues [51] reported a lack of change in levels of cortical and subcortical TSPO tracer uptake for two years, despite the clinical deterioration of PD patients. Similarly, microglia TEP imaging followed-up with a second generation of TSPO radioligand (^11^C-DPA713) also failed (in ROI—Regions of interest—analysis) to show increased tracer binding at one year in earlier stage PD patients [55]. Therefore, microglial activation assessed by TSPO PET imaging seems to be relatively static over time in PD, and only microglia phenotypes may change with the disease progression. 

Multiple system atrophy (MSA) is another form of atypical parkinsonism in which overlapping symptoms as well as tremor, akinesia and rigidity are associated with autonomic failure and cerebellar dysfunction. In contrast to PD, the accumulation of α-synuclein fibrils leads to the generation of glial cytoplasmic inclusions (GCI) which affects mostly the oligodendroglia rather than neurons [59].

In MSA patients, significantly increased ^11^C-PK11195 uptake has been observed in the dorsolateral prefrontal cortex, putamen, pallidum, pons and substantia nigra, reflecting the known distribution of microglial and neuronal lesions in MSA [66]. Additionally, TSPO is overexpressed in the white matter of MSA and Progressive Supranuclear Palsy (PSP) patients [61]. In MSA, these results appear to echo the findings of neuropathological studies and could reflect the demyelination process which predominantly affects the MSA-C variant [67]. Unlike the PD scenario progression, microglial activation in a mouse model of MSA-like neurodegeneration occurred parallel to the dopaminergic neuronal loss in the locus niger [68]. Remarkably, the analysis of histopathological patterns of MSA brains revealed that the amount of GCIs and the level of microglial activity is reduced especially since the tissue injury becomes severe in white matter areas, unlike gray matter areas [69]. If we generalize this concept of microglial (down/up)-regulation according to lesion severity in MSA, that could explain that ^11^C-PK11195 binding increased in the thalamus or decreased in the substantia nigra after 24 weeks of active disease, compared to baseline [63].

Apart from α-synucleinopathies, tauopathies such as PSP and corticobasal degeneration (CBD) belong to parkinsonian disorders. In addition to parkinsonism, the clinical features of PSP include early falls and abnormal eye movements (vertical supranuclear gaze palsy) [70]. The hallmark of PSP comprises the deposition of 4-repeat (4R) tau protein in neurofibrillary tangles and neuropil threads that are found in the basal ganglia and brainstem [71].

In vivo microglia imaging showed increased ^11^C-PK11195 binding in basal ganglia, thalamus, midbrain, substantia nigra, pons, cerebellum and frontal lobe in PSP patients [51]. A post mortem study by Fernández-Botrán et al. [72] specified that the high degree of correlation between microglial activation, tau burden and the areas of degeneration (substantia nigra and subthalamic nucleus) previously described [73], involves a high expression level of IL-1β. Although the involvement of microglial activation as etiology or consequence remains unclear, these results suggest that there is an M1 reactive phenotype of microglial cells at a point of PSP progression. Similar to PD, while PSP patients clinically declined over 6 to 10 months, there were no significant changes in TSPO PET imaging [51].

CBD is a rare neurodegenerative tauopathy in which the hyperphosphorylated 4R tau deposits concern neurons and glia, in both the cerebral cortex and the basal ganglia. In CBD, a recent report supports that astrogliopathy precedes neuronal loss, which leads to clinical symptoms as well as asymmetrical akinetic-rigidity, apraxia, disequilibrium and limb dystonia [74]. Elevated ^11^C-PK11195 binding in the striatum, thalamus and substantia nigra [64,65] is in consonance with neurological injury patterns in CBD [73,75], especially tau load [74]. Besides the diagnostic interest of PET, molecular imaging has also been used as a tool to evaluate treatment efficacy in neurodegenerative disorders [60,62,63]. 

### 3.2. Huntington’s Disease

Huntington’s disease (HD) is an autosomal dominant inherited trinucleotide (CAG) repeat neurodegenerative disorder in which the age at onset is inversely correlated with the size of the polyglutamine repeat expansion [76]. This feature allows for the dichotomization of HD patients into premanifest HD, i.e., gene carriers are clinically asymptomatic, and manifest HD, that is to say, after motor onset of the disease [77]. This mutation in the exon 1 of the gene encoding the Huntingtin protein (HTT) leads to a mutant HTT (mHTT) which is expressed by peripheral monocytes of premanifest HD patients [78], and induces both a loss of function (e.g., regulating intracellular transport, controlling apoptosis pathway) and a gain of toxic functions (e.g., abnormal protein-protein interaction, promoting pro-inflammatory cytokines) [79,80]. HD is neuropathologically characterized by the formation of intranuclear inclusions of mHTT [81] resulting in striatal, cortical and hypothalamic atrophy [82,83] which are associated with an accumulation of reactive microglia in these same regions [31,84]. In line with these findings, in vivo microglia PET imaging showed an overexpression of TSPO in the striatum [84,85,86,87,88] ,cortical regions [85,87,88,89] and hypothalamus [87,88] (Table 3). According to lesion topology, which even depends on the stage of HD, symptoms include motor troubles (chorea, parkinsonism), cognitive decline (slowed mentation, impaired strategy) and psychiatric disorders (mood, irritability, apathy, depression) [77,90]. To such an extent, microglial activation seems to begin in cognitive regions, especially in associative striatum and correlated with cognitive dysfunction [88]. Moreover, striatal 11C-PK11195 binding is strongly correlated with the five-year probability of disease onset in asymptomatic HD gene carriers [86,88]. Furthermore, in manifest HD patients, the correlation between striatal TSPO tracer binding and the severity of motor symptoms (evaluated by the unified Huntington’s disease rating scale) [85,88] could echo the ex vivo relationship found between neuronal loss and density of reactive microglia [84]. Although HD is a monogenic disease, recent studies revealed that different genes, distinct from the HD locus itself, could change disease manifestation and progression [91]. We hypothesize that these genetics and thus clinical features could explain the difference in microglial activation, objectified by ventral striatum TSPO tracer binding variability in premanifest HD patients [88,89].

### 3.3. Dementia

Alzheimer’s disease (AD) is the most prevalent neurodegenerative cause of dementia and is characterized by amnesic memory impairment, language deteriorate and behavioral changes due to cognitive decline [92]. The neuropathological hallmarks of AD include extracellular peptide amyloid β (Aβ) forming neuritic plaques and neurofibrillary tangles (NFTs) of intraneuronal deposits of hyperphosphorylated/misfolded tau proteins. Each of these lesions has a characteristic distribution during the evolution of AD and has a more or less strong clinicopathological correlation. A transcellular propagation of abnormal tau proteins [93] and Aβ [94] occurred in AD. Regarding the neurofibrillary degeneration, it starts in the entorhinal cortex and then spreads to synaptically connected brain areas such as the hippocampus/limbic system, and finally affects the frontal and parietal lobes (i.e., associative isocortex) [95]. The amount and the topography of these NFTs match with the neuronal loss and correlate with the severity and the duration of dementia as well as lesions topology (e.g., neocortex, limbic system) is in consonance with the clinical features of AD (e.g., cognitive impairment) [96]. Moreover, tau hyperphosphorylation seems to be promoted by a pro-inflammatory environment (e.g., IL-6 release [97]) and may support microglial activation [98], highlighting the vicious circle of tauopathy’s process in AD (for review, see Calsolaro et al., 2016 [99]). Microglia PET imaging supported these experimental results. Indeed, microglia PET imaging revealed greater radioligand uptake in AD patients than in healthy controls in the same regions which are sequentially affected by neurofibrillary degeneration: entorhinal cortex [100,101,102], hippocampus [101,103,104], frontal [103,104,105,106] and parietal cortex [100,101,103,104,105,106,107,108,109,110] (Table 4). Longitudinal studies exhibited an increase in cortical-activated microglia during the course of AD [101,103,110]. Similarly, cross-sectional post mortem studies using the temporal neocortex of 40 AD subjects with a symptom duration ranging from 4 to 20 years showed a significant positive association between CDC68+ microglia and symptom duration [101]. Comparison in TSPO density between mild cognitive impairment due to AD (MCI) and AD dementia yielded ambiguous results: significant difference was found between the two groups [101,102,109] or was absent [110,111,112] according to different studies. MCI represents a diagnostic entity for the transition between normal aging and AD dementia [113]. Analogous to Aβ and tau that has already accumulated in the brain of MCI patients [114], PET imaging showed that microglial activation occurred before the onset of clinical symptoms of dementia [108,110,115]. These results are consistent with the presence of neuroinflammatory biomarkers in the cerebrospinal fluid of pre-symptomatic AD subjects [116]. Nevertheless, some studies provided contradictory results [109,111,112].

Unlike NFTs, amyloid plaques deposition showed a lesser stereotypical spatiotemporal pattern of progression; they accumulate mainly in the isocortex, and the amyloid burden does not correlate with the severity or the duration of dementia [117,118]. Supported by various biomarker-autopsy correlation studies, Jack et al. [119] proposed that AD biomarker curves assume a sigmoidal shape as a function of time, involving a threshold effect and a late deceleration of tau and Aβ loads. With current data on microglial activation, it seems difficult to give a similar trend to the progression of neuroinflammation in AD dementia. However, microglial activation states obviously play a significant role in aging and age-related disease, especially in AD. Aβ (both, soluble [120] and fibrillary [121]) can bind to several Toll-like receptors (TLR) and co-receptors expressed on microglia, leading to its activation [122] and promoting the formation of clusters of activated microglia surrounding amyloid plaques in AD brains [123]. In line with these ex vivo findings, TSPO PET imaging showed higher microglial activation in various cortical regions known to be the seat of Aβ deposits [124] (e.g., prefrontal [104,107,108], temporal [100,101,102,104,105,106,107,108,109,125], occipital [101,104,105,107,108,125], precuneus [101,109,110], cingulum [100,104,110]) in AD dementia patients, compared to healthy subjects. This Aβ-induced inflammatory environment [126], combined with effect of immunosenescence on microglia [127,128], lead to a situation in which microglia that initially exhibited a neuroprotective M2 phenotype at the beginning of AD [129], become confined to a classical activated state (i.e., M1) with strongly reduced Aβ-clearing capabilities in the advanced stage of AD [7]. 

Correlation analysis between TSPO tracer binding and AD severity provided heterogeneous findings. In most studies, in parallel to cognitive deterioration, objectified by a decreased MMSE score [105,106,125] or increased CDR-SB [101,109], TSPO expression density in cortical regions was linearly raised. To the same extent, Kreisl et al. [101] exhibited that early onset AD (EOAD) patients presented significantly higher ^11^C-PBR28 than later onset AD (LOAD) patients, both at baseline and follow-up. This overexpression of activated microglia in more active AD (i.e., early onset) may confer on it the status of a deleterious process. Other studies found no link [105,108,109,113]. Conversely, one of the surprising results of the study of Hamelin et al. [110] is that MMSE scores correlate positively with TSPO tracer binding. Moreover, the radioligand (^18^F-DPA-714) GCI was greater in slow decliners than in fast decliners, suggesting an early neuroprotective effect of microglial activation in AD. 

In light of such antipodic findings, we cannot exclude the fact that TSPO PET imaging has limited abilities to robustly reflect microglial activation in AD. In fact, Banati et al. [33] suggested that the overexpression of TSPO in activated microglia may be related to structural changes in mitochondria due to morphological changes in microglial cells. On the basis of that postulate, we hypothesize that the profound morphological deterioration of microglial cells which occurred in AD [128] could be a source of signal variability in TSPO PET imaging.

Frontotemporal lobar degeneration (FTLD) is a clinically, pathologically and genetically heterogeneous syndrome characterized by progressive clinical features such as behavioral and personality changes, cognitive decline and parkinsonism [137]. These symptoms result from progressive neurodegeneration and, ultimately, focal atrophy of the frontal and temporal cortex in which inclusions of hyperphosphorylated tau protein in neurons and glial cells (FTLD-tau) and inclusions of TDP-43 (FTLD-TDP) compose the two main histopathological hallmarks of this disease [138]. FTLD has a strong genetic component; mutations causing FTLD include MAPT (microtubule-associated protein tau) and progranulin [139]. Interestingly, proganulin is expressed by activated microglia [140] and polarizes microglial cells into M2 anti-inflammatory phenotypes [130], suggesting that microglia polarization deficiency could play a role in FTLD. Indeed, Cagnin et al. [133] demonstrated an enhanced uptake of tracer (^11^C-PK11195) in the typically affected frontotemporal regions. Two MAPT pre-symptomatic gene carriers exhibited microglial activation in either, posterior cingulate cortex, region which sends projection to the superior temporal sulcus, or in the medial frontal cortex [131]. Elevated putaminal uptake of TSPO tracer in frontotemporal dementia patients [130] is consistent with the neuropathological (involvement of the basal ganglia [141]) and clinical features (parkinsonism [142]) of FTLD.

### 3.4. Amyotrophic Lateral Sclerosis

Amyotrophic lateral sclerosis (ALS) is a rare adult-onset neurodegenerative disorder in which upper and lower motor neurons degenerate leading to progressive muscle paralysis then respiratory failure with a fatal outcome, typically within 2–4 years from onset [143]. This motoneuropathy that occurred from 5% to 20% in a positive family history of ALS, fronto-temporal demence (FTD) or both, results in fasciculation, cramps, muscle atrophy associated with hyperreflexia and spasticity [144,145]. Central inflammation has been proposed to play a role in the motor neuron death process in ALS [146]. TSPO PET imaging supported this assumption. Indeed, ALS patients presented significantly greater TSPO tracer binding in motor cortex than healthy volunteers [32,147,148]. Moreover, “microgliopathy”, assessed by CD68- and Iba1-reactive microglial cells, correlated with the disease progression and is linked to the severity of upper motor neuron deficits in the corticospinal tract of ALS patient autopsies [149]. These ex vivo findings were clinically sustained by PET studies, where TSPO expression density was correlated with motor symptoms severity, assessed by the UMND (Upper Motor Neuron Disorders) score [32,147] (Table 5). Interestingly, the one study that described an elevated TSPO radioligand uptake (^11^C-PBR28) in the brainstem of bulbar-onset ALS patients was also the one in which a statistical correlation was found between SUVr values and ALSFRS-R (revised ALS functional rating scale) [147]. Given the fact that this functional scale is more accurate to evidence lower motor neuron involvement [32], this association is probably due to the fact that microglial activation in the brainstem could reflect inflammation around the lower motor neurons of the cranial nerves [147]. The murine model of ALS, in which microglial cells overexpressed mutant-superoxide dismutase (mSOD1), the main cause of non-sporadic ALS, point out the pathogenic role of microglia in ALS; mutant protein may be more involved in disease progression than in its initiation [150]. Inversely, activated microglia in the temporal region could be involved in the early stage of ALS [148], and then progressively decrease. Indeed, this cerebral area was not affected in ALS patients with a more advanced stage [32,147]. Like AD dementia, a switch from the classically activated M1 phenotype to the alternatively activated M2 microglial state takes place during the progression of ALS disease [151,152].

### 3.5. Psychiatric Disorders

Bipolar disorder (BD) is a chronic mood trouble in which episodes of elevated mood and manic state are typically alternated with major depressive access, interspersed by euthymic periods [153]. Although the pathophysiology of this severe mental illness remains to be elucidated, a large body of literature suggests that several pathophysiological processes may sequentially and/or concurrently occur in BD, as well as corticolimbic control circuits deterioration, monoaminergic neurotransmission imbalance or microglia homeostasis disruption (for review, see Sigitova et al., 2016 [154]). Among these, hippocampal neuroinflammation, assessed by enhanced ^11^C-PK11195 binding compared to healthy subjects [155], could echo the change in the cytokines pattern’s profile (TNF, IL-1, IL-2 and IL-6) of BD-I patients [156] (Table 6).

Major depressive disorder (MDD) is a highly prevalent condition, affecting nearly 4% of the adult population [157], which could complicate neurodegenerative disorders (e.g., MS [158], PD [159]) or be isolated, without comorbidities. Although this depressive disorder mainly concerns mood disturbance, clinical features include a large range of symptoms as cognitive deficits, vegetative impairment (e.g., loss of appetite, sleeping difficulties) or behavioral deterioration (e.g., anhedonia, suicidal ideation). Several data, such as the association between central inflammation and depression-like symptoms in rodents [160], abnormalities in peripheral cytokines of depressed patients [161], or association between interferon alpha in the context of hepatitis C and depressive symptoms [162], have led some investigators to propose a microglial dysregulation theory for MDD etiology [163]. Nevertheless, post mortem studies have questioned this assumption. Indeed, no evidence of microglial activation was found in the brain of MDD patients [164,165,166]. In line with these results, microglia PET imaging did not reveal any statistical difference in ^11^C-PBR28 binding between healthy volunteers and patients with mild to moderate depression [167]. However, Setiawan and colleagues [168] recently found increased microglial activity (^18^F-FEPPA, V_T_, total distribution volume) in the anterior cingulate cortex of MDD patients, the region known to be implicated in the modulation of emotional behavior [169]. Moreover, this PET imaging result was correlated with the MDE (major depressive episode) severity, objectified by a 17-item Hamilton depression rating scale (HDRS) score [168]. 

Apart from epidemiological [178] and genetic [179] evidences of overlap between BD and schizophrenia (SCZ), molecular imaging of microglia of these psychiatric disorders also share some similarities, especially hippocampal TSPO overexpression compared to normal controls [155,172]. SCZ is a chronic early adult-onset psychiatric disorder in which symptoms are typically divided into positive (e.g., hallucinations), negative (e.g., affective flattening, alogia) and cognitive (e.g., concentration and memory impairment) [180]. Multiple lines of evidence from immunohistochemical cell counting studies to clinical trials of a non-selective microglial inhibitor support the fact that microglia is involved in the pathophysiology of SCZ [181]. Nevertheless, as with post mortem studies [182], microglia PET studies provided conflicting results. Indeed, some studies revealed significantly higher TSPO radioligand binding in SCZ patients in total gray matter [171,175], especially in the frontal and temporal lobes [175] and the hippocampus [172] than in controls subjects. However, other studies found no clear difference between normal controls and SCZ patients in various brain regions [173,174]. 

Neuroinflammation imaging in psychiatry is in an early stage, and there is much more to investigate (for review, see Slifstein and Abi-Dargham, 2016 [183]), especially regarding TSPO imaging. Indeed, there is evidence that the dysregulation of steroidogenesis by TSPO is associated with psychiatric disorders. For instance, an association has been found between rs6971 polymorphism in the TSPO gene and the diagnosis of BD [184].

### 3.6. Multiple Sclerosis

Multiple sclerosis (MS) is a chronic autoimmune disease of the CNS in which migration of myelin-reactive T-cells into the CNS is followed by astrocyte and microglial activation, recruitment of peripheral macrophages and oligodendrocyte destruction, resulting in focal and diffuse demyelination [185,186]. The clinical presentation of MS as well as symptoms produced by the episodes of neurologic dysfunction (i.e., “attacks” or “relapses”) are heterogeneous. Regarding the symptoms of MS, tingling, weakness, vision loss, incoordination or bladder dysfunction may occur and depend upon the site of neurologic involvement. As regards the clinical picture, the most typical form at the onset of the disease is relapsing-remitting MS (RRMS), which is characterized by attacks interspersing with neurologically stable periods. Over several years to decades, RRMS shifts into secondary progressive MS; the recovery between relapses becomes partial and gradually provokes persistent symptoms which lead to irreversible disability. Only a minority of patients (~10–20%) experience insidious worsening without acute attacks, a clinical course known as primary progressive MS (PPMS) [187,188]. The major substrates of the disease progression include demyelinated cortical lesions, diffuse cortical and deep gray matter degeneration [189], which are associated with the chronic activation of microglia [190,191,192]. Microglia play a central role in maintaining the central chronic inflammation in MS [193,194], to the extent that its presence and distribution in the plaques allow for the neuropathological classification of the nature of the white matter lesions (i.e., active versus inactive) in secondary progressive multiple sclerosis (SPMS) [195]. 

Although the clinical and/or MRI discovery of dissemination of the lesions in time and space remains the cornerstone of MS diagnostics, TSPO PET imaging could be a promising technique to diagnose MS or to detect the conversion of RRMS to SPMS. In light of the limits of clinical assessment (e.g., measuring EDSS, expanded disability status scale) [196] and MRI parameters [197] to predict the progression of MS, TSPO PET studies have shown hopeful findings (Table 7). Indeed, activated microglia in SPMS relative to RRMS are characterized by both specific and more diffuse distribution in cortical regions [198] as well as greater TSPO radiopharmaceutical uptake, especially in the thalamus [199,200] and deep gray matter [200]. Provided that these results will be replicated in larger cohorts, it paves the way to early intervention in MS, thanks to TSPO PET imaging, in order to prevent or efficiently treat SPMS. At the upstream level in the disease history, Giannetti and colleagues demonstrated in clinically isolated syndrome (CIS), the prodromal state of MS, that patients who developed MS at two years had higher ^11^C-PK11195 binding at baseline, especially in normal-appearing white matter (NAWM) [201]. Analogous to the relationship between MCI and AD, we hypothesize that NAWM TSPO tracer uptake of CIS subjects could allow for the establishment of an *x*-year probability of disease (i.e., MS) onset. This significant increase in NAWM TSPO density, compared to healthy volunteers, has also been found in RRMS [200] and SPMS patients [200,202]. In addition to detecting activated microglia in areas of the brain which appear normal in MRI, the NAWM, TSPO PET imaging could predict the development of some gadolinium-enhancing lesions. In fact, increased focal ^11^C-PBR28 binding in an area of NAWM at the time of the PET scan corresponded to a contrast-enhancing lesion at a follow-up MRI exam one month later, as was found in two MS patients, suggesting a role for early microglial activation in MS lesion formation [203]. Apart from the fact that microglial activation may precede MS lesions, and that there is a greater TSPO radioligand uptake in RRMS than in SPMS, a body of evidence gives a deleterious status of activating microglia in MS. Among these, RRMS patients who had the greatest number of relapses (i.e., more clinically active) also had the highest ^11^C-PK11195 binding potential [198,204]. Furthermore, association analysis revealed that TSPO tracer binding levels in various brain regions correlated positively with both MS score severity (e.g., EDSS) [198,200,205,206] and disease duration [199,203]. This set of results is in consonance with post mortem studies in which severe cortical microglia activation and demyelination are associated with a pejorative outcome [207,208,209]. Nevertheless, some microglia PET studies provide conflicting results in terms of the association analysis in either EDSS [210,211] or disease duration [204,210].

Ratchford and colleagues [204] provided proof of the concept that microglia PET imaging with ^11^C-PK11195 could also be a tool to assess disease-modifying drugs for RRMS efficacity. Indeed, radiopharmaceutical binding potential per unit volume was statistically decreased in the whole brain after one year of glatiramer acetate. This result supported the in vitro evidence of its mechanism of action in which an inhibition of transformation to an activated microglia form could be responsible for therapeutic effects [212]. 

## 4. Multiplex Approaches to Improve TSPO PET Imaging Significance

TSPO molecular imaging provides valuable information such as presence, intensity and localization of the microglia activation, but this uniparametric approach might be considered as one component of a multiplexed strategy. Indeed, TSPO imaging combined with further PET targets, imaging techniques (e.g., MRIf), or even biology, could allow for a deeper understanding of the role of activated microglia in neurodegenerative disorders. We organize this section into; first, unimodal approaches, that is to say, TSPO PET imaging coupled with other PET radiopharmaceuticals; and second, multimodal approaches associating microglia PET imaging with other modalities.

### 4.1. Unimodal Approaches

#### 4.1.1. Neuroinflammation PET Imaging Targeting Molecular Biomarkers Other than TSPO

Neuroinflammation is a complex physiological response to CNS injuries involving a biochemical molecular cascade involved in both tissue degradation and remodeling. Activation of microglia cells that leads to cell death is often related to oxidative stress through cytokines and chemokines release, glutamate-induced excitotoxicity, mitochondrial dysfunction or by protein aggregation. As described above, PET studies exploring neuroinflammation in CNS disorders have mainly been focused on the evaluation of the sole TSPO. Therefore, identifying and imaging other molecular biomarkers involved in inflammatory cascade would be a promising approach combined with TSPO imaging.

PET imaging associating TSPO radioligands with tracers for other molecular targets involved in microglial activation has already allowed for the assessment of temporal progression of different biochemical mechanisms involved in neuroinflammation. Indeed, in the rodent model of cerebral ischemia, Martín et al. have assessed in parallel the in vivo expression of both α4β2 (nicotinic acetylcholine receptors) and TSPO in reactive glial cells using 2-^18^F-fluoro-A85380 and ^11^C-PK11195, respectively [219]. Results have shown a progressive binding increase during the first week after cerebral ischemia, followed by a progressive PET-binding decrease in the ischemic territory. Likewise, differential time-dependent activation of TSPO and matrix metalloproteinases (MMPs), protease involved in tissue degradation and remodeling has been observed by PET co-imaging with the ^18^F-DPA714 and the ^18^F-BR-351, suggesting that their expression after stroke are subsequent or independent phenomena [220]. While TSPO is known to be an early biomarker in the neuroinflammation cascade, another longitudinal investigation, using ^18^F-FSPG and ^18^F-DPA-714, in the cerebral ischemia model displayed an earlier expression of the cystine/glutamate antiporter (system xc-), involved in glutamatergic excitotoxicity, compared to TSPO imaging, evidencing its novel role on inflammation after cerebral ischemia [221]. ^18^F-FSPG, a system xc- PET tracer, combined with ^11^C-PK11195 has also been used during the course of experimental autoimmune encephalomyelitis (EAE) induction in rats. In this MS model, ^18^F-FSPG showed on the one hand a significant increase of system xc-function in the lumbar section of the spinal cord at 14 days but, on the other hand, showed a significant increase of microglial/macrophage activation in the spinal cord and cerebellum two weeks after EAE induction. These dissimilar binding profiles between both PET tracers might be due to the existence of different microglial/macrophage populations throughout the CNS following MS [222]. In the same way, to assess COX implication during the neurodegenerative process in AD, COX-1 and TSPO PET co-imaging provided details of the time course in the inflammatory process in a rodent model of neuroinflammation (induced by intrastriatal injection of lipopolysaccharide or quinolinic acid). High accumulation of COX-1 tracer (^11^C-ketoprofen methyl ester) was observed in the early phase, matching with the time course of microglial activation and macrophages, whereas the high accumulation of ^11^C-PK11195 started slowly and lasted until at least 14 days afterward, corresponding to changes in both the activation of microglia/macrophages and astrocytes [223].

Thus, upregulated biomarkers in reactive glial cells complementary to TSPO are expected in order to fulfill the current clinician’s expectancies regarding the microglial role in neurodegenerative diseases and associated potential novel therapeutic approaches.

#### 4.1.2. Dopaminergic PET Imaging

Dopaminergic neurotransmission is a key player in various brain functions, including behavior and motor functions, and is involved in the pathogenesis of psychiatric (e.g., SCZ, depression) and neurological disorders (e.g., PD, parkinsonian syndromes) [224]. Movement disorders are strongly linked with dopaminergic impairment of the striato-pallido-thalamic-cortical axis in which the striatum receives afferent projections from cortical glutamatergic and nigral dopaminergic neurons [225]. Molecular imaging of dopaminergic presynaptic integrity (e.g., DAT with ^11^C-CFT or ^18^F-FE-PE2I), dopamine synthesis and turnover (e.g., ^18^F-DOPA) and postsynaptic dopamine receptors (^18^F-Fallypride) are used to evaluate this dopaminergic pathway in various CNS conditions [224]. Experimental models of PD suggest that microglial activation may lead to dopaminergic neuronal loss though iNOS-dependent oxidative stress [226]. Three clinical studies have been performed to assess this association between microglial activation and dopamine terminal loss in PD [49,51,62]. Concerning PD patients under antiparkinsonian medication, association analysis revealed that microglial activation did not correlate significantly with either putaminal ^18^F-DOPA signal [65] or nigrostriatal DAT levels, objectified by ^18^F-FE-PE2I binding [62]. Nevertheless, the mechanism of action of antiparkinsonian drugs is based on the enhancement of dopaminergic transmission in the brain, therefore in this situation PET imaging of the dopaminergic system is de facto biased, as previously demonstrated [227,228]. The one study conducted on ten drug-naive patients with PD (rated at stage 1 to 2 on the Hoehn and Yahr scale) brought out the fact that ^11^C-PK11195 binding in the midbrain significantly and negatively correlated with those for ^11^C-CFT in the dorsal putamen, a region known to engage in motor execution [49]. Moreover, this microglial activation correlated positively with the motor severity (UPDRS assessment) [49], suggesting a causal deleterious relationship between microglial activation and nigrostriatal pathway impairment, both clinically (UPDRS) and biochemically (central hypodopaminergism). However, this association does not elucidate the sequence of events. Indeed, dopaminergic neuronal loss is accompanied by the release of endogenous constituents known as damage-associated molecular patterns (DAMPs), which are able to activate microglial cells [229]. 

#### 4.1.3. Abnormal Protein Aggregates PET Imaging

Amyloid deposition, tangle formation, activated microglia and neuronal dysfunction are pathological processes involved in AD. However, the relative role of these processes in driving disease progression is still unclear. As stated above, microglia PET imaging in AD provides heterogeneous findings, hence the association analysis between fibrillar β amyloid, objectified by ^11^C-PIB binding, and microglial activation could (i) investigate longitudinal changes of microglial activation and amyloid; and (ii) assess the temporospatial relationship between these processes in AD. Indeed, a positive correlation was found in most of the cortical regions between amyloid burden and microglial activation [103,110,217]. Longitudinal study revealed that this correlation was less pronounced at follow-up, and the location of the correlated regions presented a dynamic change [103], suggesting that microglial activation is not static and its progression during AD may fit with the model of Calsolaro et al. [99]. Furthermore, the positive correlation between amyloid load and microglial activation is stronger in AD patients than in MCI patients, suggesting that microglial activation could be driven by the presence of amyloid deposition [103] while other studies challenged these findings in MCI patients [111,115]. Likewise, a positive correlation was seen in the parietal lobe and the anterior cingulate cortex in PDD patients [125]. This combined approach of amyloid and microglial imaging is nevertheless submitted to debate by some studies pointing out no correlation [101,105,109,111] or negative association with amyloid deposits in the posterior cingulate cortex in AD patients [106]. 

On the same model as the amyloid/microglia imaging, with the emergence of new NFTs PET tracers, the combined use of tau and microglial imaging should also contribute to the pattern of distribution of the increased tau deposition and widespread microglial activation in AD subjects. Not only because post mortem and initial tau imaging studies indicate that the amount of tau deposition and their topographical distribution in the brain might be more relevant, and more tightly associated with neurodegeneration and cognitive decline than β amyloid deposition [114], but also because in studies of tau model mice using ^18^F-FEDAA1106 or ^11^C-Ac-5216, microglial TSPO expression was linked to neurotoxic tau pathology. Nevertheless, to our knowledge, no combined use of tau and microglial imaging study has been performed to date.

Furthermore, recent studies suggest that neuroinflammation and α-synuclein disorders are interconnected processes which promote each other and perpetuate neurotoxicity. This interaction leads to distinct microglial polarization states and drives the chronic progression of neurodegeneration [230,231]. Thus, it seems appropriate to combine neuroinflammation/α-synuclein imaging, for which efficient radiopharmaceutical are currently strongly expected.

#### 4.1.4. PET Tracer in MS

Microglia PET imaging (^11^C-PK11195) has been used in combination with ^11^C-methionine for the differential diagnostics between tumefactive MS lesion and low-grade glioma in a case report [231]. Beside this diagnostic potential of TSPO PET imaging in MS as previously described, the hopeful results of myelin PET tracers in animal studies such as ^11^C-CIC or ^11^C-MeDAS (for review, see Faria et al. [185]) lead us to believe that a multiplexed approach with these radiotracers could allow us to understand the relationship between the demyelination/remyelination process and microglial activation in MS.

#### 4.1.5. Cerebral Metabolic State Imaging: ^18^F-FDG

Cerebral glucose metabolic rate (rCMRGlc) measured by ^18^F-fluorodeoxyglucose (^18^F-FDG) PET is currently the most accurate in vivo method for the investigation of regional human brain metabolism, and is suggested to be to be an indirect measure of synaptic function [132]. Thus, changes in neuronal activity induced by neurodegenerative diseases are reflected in an alteration in glucose metabolism.

A significant negative correlation between microglial activation and glucose metabolism was present in the hippocampus [125,132], medial temporal cortex [125] in AD patients, in the temporoparietal and occipital cortex [125] and hippocampus [132] in PDD patients, and in the hippocampus in MCI patients [125] . This inverse correlation implies the direct damaging effect by the activated microglia on neuronal loss in AD and PDD, objectified by the glucose metabolism decrease. In addition, Fan and colleagues have used three combined molecular imaging radiopharmaceuticals (^11^C-PIB, ^11^C-PK11195 and ^18^F-FDG) to show a correlation between amyloid load, levels of microglial activation, and reduced glucose metabolism in AD and, to a lesser extent, in MCI and PDD subjects; furthermore, the correlation between amyloid load and glucose metabolism was more extensive at follow-up and was consistently associated with cognitive decline in AD patients, suggesting that, once synaptic dysfunction occurs, the disease is active and progressive [85]. 

### 4.2. Multimodal Approaches

#### 4.2.1. Imaging Modalities Other Than PET

In order to investigate CNS disorders by a broader approach combining concomitantly complementary pathophysiological aspects, the use of TSPO PET in a multimodal neuroimaging strategy has increased significantly in recent years. In this framework, MRI and its different subtypes (i.e., structural, diffusion and functional MRI) are among the most democratized approaches, in combination with TSPO PET imaging.

Brain atrophy assessed in structural MRI has been found in various CNS disorders [232]. This is especially true in AD, where hippocampal atrophy correlates with clinical decline and assumes the status of both the diagnostic and imaging biomarker of AD progression [233]. This approach was used to assess the relationship between microglial activation and neurodegenerative process in AD [101,124], PDD [132], SCZ [177] and MS [200,205,213,218]. Indeed, the amount of activated microglia in the whole cortex, medial temporal lobe and hippocampus was inversely correlated with hippocampal volume and ^18^F-FDG hippocampal uptake in AD and PDD patients [132]. In front of this spatial agreement between microglial activation in the cortical projections from the hippocampus and hippocampal atrophy, the authors assumed that microglial cells in a reactive state might be one of the effectors of neurodegeneration [132]. The same association between TSPO density increase and cerebral volume decrease reached significance in various cortical regions such as the inferior parietal lobule, precuneus, superior temporal cortex and especially in the prefrontal cortex [101], in which interconnection with the thalamus and hippocampus [233] are strongly linked to episodic memory impairment in AD patients [234]. On the contrary in BD-I patients, association analysis between ^11^C-PK11195 binding potential and hippocampal volume failed to exhibit a significant relationship [170]. Quantitative magnetization transfer imaging (qMTI), an advanced structural MRI technique providing information about in vivo tissue integrity, is able to detect inflammation-related changes in brain microstructures [235]. Furthermore, qMTI in MS patients has shed light on the pattern of tissue structural abnormalities in NAWM being greatest near the ventricles [236]. In a way to characterize the temporal/sequential/spatial relationship between microglial activation and anatomically objective brain lesions, a coupling TSPO PET/qMTI could be a promising approach, especially in MS.

Diffusion-weighted imaging, reflecting (ab)normal tissue diffusivity (measured by apparent diffusion coefficient (ADC)), has been used in combination with ^11^C-PK11195 in PD, MSA and PSP patients [61]. Although the small sample size limits the scope of study’s conclusions about the impact of microglial activation on tissue damage, a positive correlation between microglial activation and ADC was found in the pons of atypical parkinsonism patients. Given the fact that ADC indirectly reflects cellularity, this association suggests a causal relationship between microglial activation/TSPO overexpression and gliosis.

fMRI is a technique that uses blood-oxygen-level-dependent (BOLD) contrast to detect changes in brain hemodynamics. Hippocampal functional connectivities to the subguenal and prefrontal and parietal regions correlated with the beck depression inventory scores and ^18^F-PBR111 DVR in MS patients. This correlation analysis between hippocampal ^18^F-PBR111 binding and functional connectivity, assessed by fRMI, lead authors to hypothesize that microglial activation is involved in MDE pathophysiology in MS patients [218]. Besides “conventional fMRI”, innovative techniques like GluCEST (Glutamate chemical exchange saturation transfer) appear to be hopeful tools to assess and understand neuroinflammation [237], and may be even more useful in a multiplexed approach with microglia PET imaging.

Beyond the interest of coupling MRI techniques with TSPO PET modality, recent advances in imaging of activated microglia have been shown promising results. Indeed, 2D optical imaging spectroscopy [238] or optical projection tomography applied to a 3D spatial assessment of the development of neuroinflammation [239] provided positive outcomes in rodent models.

#### 4.2.2. Biology

The understanding of the microglial activation status in neurodegenerative disorders could be reached by coupling microglia PET imaging to several biological techniques including metabolomics, transcriptomics, magnetic resonance spectroscopy (MRS) and measurement of central and/or peripheral pro-inflammatory components. Among these, ^1^H MRS in BD-I patients provided surprising results. Whereas no relationship was found between phosphocholine + glycerophosphocholine concentrations, cellular turnover biomarkers and microglial activation; *N*-acetylaspartate + *N*-acetyl-aspartyl-glutamate concentrations, representing loss of neuronal integrity when levels decreased, were positively associated with ^11^C-PK11195 binding in the left hippocampus [170]. These findings challenged the neuroinflammation theory of psychiatric disorders in which activating microglia have a negative effect on neuronal survival [240].

In MS patients with a higher inflammatory load, the concentration of myoinositol, a metabolite which has been proposed as a glial and especially as an astrocyte marker [241], was correlated with ^11^C-PBR28 DVR in WM lesions, suggesting the joint activation of astrocytes and microglia in MS [211]. 

The association between inflammatory biomarkers (cytokine levels [89,168], or proteins carbonylation [54] and TSPO PET imaging in neurodegenerative disorders was also the subject of several studies. Early stage dementia with Lewy bodies (DLB) and PD patients exhibited significantly greater levels of total carbonylated proteins in cerebrospinal fluid (CSF) than healthy controls, suggesting that relative aspecific oxidative modifications occurred in both neurodegenerative disorders [54]. The lack of correlation between central inflammation measured by ^18^F-FEPPA V_T_ and levels of several pro-inflammatory cytokines such as IL-1β , IL-6 and TNF-α supports the idea that microglial activation can occur in MDE even when peripheral inflammation is absent [168]. In patients with recent onset of SCZ, the absence of this association has also been found between ^11^C-DPA713 V_T_ and both plasma and cerebrospinal fluid IL-6 levels [176].

## 5. TSPO PET Imaging Potential Added Value in the Clinical Practice

As discussed above, TSPO PET imaging has been used in a large number of studies concerning neurodegenerative diseases over the past 20 years. However, such a major investment has not yet resulted in an expected impact for clinical practice. In AD or SCZ, for instance, our present analysis came to conflicting results, which are moreover based on small numbers (<100) of patients. The overall trend that could be drawn from these studies would suggest that early microglial activation could represent a beneficial response, whereas chronic exposure would induce detrimental effects by promoting neuronal death, thus contributing to the progression of the disease. 

A comparison between PET radioligands’ development, targeting TSPO and β-amyloid (Aβ) plaques, respectively, illustrates this assessment: while the first clinical uses of these radiopharmaceuticals were approximately contemporaneous, those of ^11^C-PK1195 being even much earlier than those of ^11^C-PiB and ^18^F-FDDNP (two of the first developed Aβ tracers) [242,243], several Aβ-radiopharmaceuticals are nowadays routinely used to assess amyloid-β burden in a clinical setting. Aβ-PET represents a major advance in the evaluation of patients with cognitive impairment when AD is a possible cause, and affects the diagnosis and the resulting treatment recommendations [243]. TSPO radiopharmaceuticals (^18^F-DPA714, ^18^F-FEPPA, ^18^F-PBR06, or ^18^F-GE180 being among the most promising ones), remain at the same time dedicated to specialized PET centers and restricted to research applications.

The current state of knowledge, which clearly identifies the neuroinflammation process as a constitutive part of the pathophysiology of neurodegenerative diseases, might give a new impetus for TSPO PET imaging, especially for the noninvasive and longitudinal evaluation of anti-inflammatory therapies for these diseases. For instance, Ratchford et al. recently reported a longitudinal TSPO PET study evaluating microglial activation in MS. One year treatment with glatiramer acetate reduced TSPO binding significantly in both cortical gray matter and cerebral white matter, when compared with cerebellum [204]. This promising approach gives excellent comparative data for longitudinal TSPO PET studies evaluating therapeutics in progressive diseases. Nevertheless, several drawbacks limit the ability of TSPO as a PET imaging molecular target in routine clinical practice, such as the identified polymorphism affecting the binding affinity properties of most of TSPO ligands and the TSPO multicellular expression, among others. Thus, we mentioned above the interest of TSPO imaging combined with further PET targets, but the dissimilar binding profile observed and its correlation with neuroinflammation cellular dynamics remains a major issue for understanding the pathophysiological and clinical relevance of what we are imaging.

## 6. Conclusions

TSPO PET tracers were the most widely used and allowed us to better characterize microglia-related neuroinflammation in terms of intensity, localization and static/dynamic feature of microglial activation in various neurodegenerative troubles. Recent studies challenged the paradigm that activated microglia are detrimentally involved in neurodegenerative process. Indeed, microglia seem to play a role in CNS disorders with a relative dichromatic status that occurs in a time-dependent fashion. With the benefit of hindsight over 25 years, TSPO PET imaging showed several limitations in relation to the multicellular expression of the target or its similar expression, whatever the microglia polarization state. Thus, new PET biomarkers reflecting some specific functions of activated microglia (e.g., polarization, microglial oxidative stress-related) conjugated with a multiplex strategy combining several modalities (imaging, biology) may solve clinicians’ concerns about characterizing the role of activated microglia over the progression of CNS disorders. 

Finally, the usefulness of TSPO PET imaging as a major research tool has appeared inescapable over years. Indeed, it has allowed for better understanding of the neuroinflammation process in CNS disorders, and the multiplex approach, either unimodal or multimodal, would certainly broaden the knowledge of this issue. Nowadays, however, the main challenge remains to identify the scope of TSPO PET imaging for a transfer in routine clinical use whether as a diagnostic, prognostic or therapeutic effects assessment tool.

## Figures and Tables

**Table 1:** A summary of the different generation of TSPO radiotracers from Vivash and O’Brien [43].

Chemical Class	Radioligand	Advantage	Limitation	Generation
Isoquinoline carboxamide	^11^C-PK11195	rs6971-insensitive	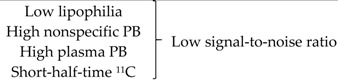	First
Indole acetamides	^11^C-SSR180575		rs6971-sensitive Reproducibility in humans Short half-time ^11^C	Second (^11^C)
Vinca alkaloids	^11^C-vinpocetine			
Phenoxyarylacetamides	^11^C-PBR28	Cerebellum validated as a reference region		
	^11^C-DAA1106			
Imidazopyridine acetamides	^11^C-DPA713			
Imidazopyridines	^11^C-CLINME			
Dihydro-9*H*-purinacetamides	^11^C-DAC			
	^11^C-AC-5216			
Phenoxyarylacetamides	^18^F-FEDAA1106	Longer half-time than ^11^C Improved signal-to-noise ratio	rs6971-sensitive Reproducibility in humans	Second (^18^F)
	^18^F-PBR06			
	^18^F-FEPPA			
Imidazopyridine acetamides	^18^F-PBR111			
Pyrazolopyrimidines	^18^F-DPA-714			
Isoquinoline carboxamide	^11^C-ER176	“rs6971—less sensitive in vivo than 2G”		Third
Tetrahydrocarbazole	^18^F-GE180	rs6971-insensitive		

PB, protein binding.

**Table 2:** PET imaging studies assessing TSPO tracers in parkinsonian disorders.

Disorder	Population	Radioligand	Main Findings	References
**PD**	10 early-stage drug-naive PD patients vs. 10 HC	^11^C-PK11195	Midbrain tracer BP_ND_ in PD group greater than in the healthy volunteers No significant difference in thalamus or striatum binding	Ouchi et al. (2005) [49]
	- 18 PD patients with a wide range of disease severity vs. 11 HC - 8 PD patients with a longitudinal follow-up (PET after 18–28 months)	^11^C-PK11195	- Significantly increased tracer BP_ND_ in basal ganglia, cortex and brainstem in PD group - Nigral and striatal levels of ^11^C-PK11195 binding remain unchanged	Gerhard et al. (2006) [51]
	14 PD patients vs. 8 HC	^11^C-PK11195	PD patients showed higher putamen and midbrain tracer BP_ND_ than controls	Bartels et al. (2010) [60]
	6 early-stage drug-naive PD patients vs. 11 HC	^11^C-PK11195	PD patients showed increased putamen and substantia nigra tracer BP_ND_ than healthy subjects	Iannaccone et al. (2013) [54]
	9 PD patients vs. HC	^11^C-PK11195	Pons tracer BP_ND_ in PD group greater than in the control group No significant difference in thalamus, striatum or midbrain binding	Kobylecki et al. (2013) [61]
	8 PD patients without dementia vs. 10 HC	^11^C-PK11195	BP_ND_ significant increase in temporal, parietal and occipital cortical regions	Edison et al. (2013) [52]
	19 PD patients vs. 17 HC	^18^F-FEPPA	No significant difference in striatal V_T_ values between PD patients and controls	Koshimori et al. (2015) [50]
	- 11 PD patients without dementia vs. 12 HC - 11 PD patients were rescanned after 1 year	^11^C-DPA713	- Significantly increases ^11^C-DPA713 BP_ND_ in brainstem, basal ganglia and occipital, temporal plus parietal cortex in PD group - In ROI analysis, extrastriatal ^11^C-DPA713 BP_ND_ remains stable	Terada et al. (2016) [55]
**PD-RCT**	24 PD patients: 18 with AZD3241 vs. 6 with placebo	^11^C-PBR28	Compared to baseline, significantly reduced V_T_ in nigrostriatal pathway, cortical regions, thalamus and cerebellum after 4 and 8 weeks of treatment No significant differences in changes of V_T_ between the two arms	Jucaite et al. (2015) [62]
**MSA**	5 MSA patients vs. 6 HC	^11^C-PK11195	Increased tracer binding in dorsolateral prefrontal cortex, putamen, pallidum, pons and substantia nigra	Gerhard et al. (2003) [51]
**MSA-RCT**	8 MSA-P patients: 3 with minocycline vs. 5 in placebo arm	^11^C-PK11195	Compared to baseline, tracer BP_ND_ decreased in caudate nucleus, thalamus, midbrain and cerebellum for 2/3 treated patients after 24 weeks of minocycline In placebo group, tracer BP_ND_ is increased in most regions after 6 months No statistically correlation between clinical markers and radioligand BP_ND_	Dodel et al. (2010) [63]
**PSP**	- 4 PSP patients vs. 7 HC - 2 PSP patients with a longitudinal follow-up (PET after 6–10 months)	^11^C-PK11195	- Basal ganglia, thalamus, midbrain, substantia nigra, pons, cerebellum and frontal lobe tracer BP_ND_ in PSP group higher than in the healthy controls - No significant change in ^11^C-PK11195 binding	Gerhard et al. (2006) [51]
**MSA/PSP**	7 MSA and 4 PSP patients vs. 7 HC	^11^C-PK11195	Increased tracer binding in putamen, thalamus, midbrain, frontal and deep white matter	Kobylecki et al. (2013) [61]
**CBD**	4 CBD patients vs. HC	^11^C-PK11195	Significantly increased tracer BP_ND_ in striatum, substantia nigra, pons and cortical regions	Gerhard et al. (2004) [64]
	1 patient with 3 year-history of CBD	^11^C-PK11195	Marked retention of tracer in posterior putamen, thalamus; moderately in pallidum, substantia nigra and caudal pons	Henkel et al. (2004) [65]

BP_ND_, binding potential; CBD, corticobasal degeneration; MSA, multiple system atrophy; MSA-P, multiple system atrophy Parkinson-type; HC, healthy control; PD, Parkinson’s disease; PSP, progressive supranuclear palsy; RCT, randomized controlled trial; ROI, region of interest; V_T_, total distribution volume.

**Table 3:** PET imaging studies assessing TSPO tracers in Huntington’s disease.

**Population**	**Radioligand**	**Main Findings**	**References**
11 manifest HD patients vs. 10 HC	^11^C-PK11195	Significantly increased tracer BP_ND_ in striatum, pallidum, frontal and parietal cortical areas in HD group Significant correlation between striatal ^11^C-PK11195 BP_ND_ and motor symptoms severity	Pavese et al. (2006) [85]
11 premanifest HD patients vs. 10 HC	^11^C-PK11195	Striatal and cortical (frontal, parietal, temporal, occipital) tracer BP_ND_ greater in HD patients than in healthy subjects	Tai et al. (2007) [86]
10 premanifest HD patients, 9 manifest HD patients and 10 HC	^11^C-PK11195	Tracer uptake is significantly higher in hypothalamus and striatum in premanifest and manifest HD than in healthy volunteers No significant difference in binding between premanifest and manifest HD No significant relationship between motor symptoms and ^11^C-PK11195 BP_ND_ in hypothalamus	Politis et al. (2008) [87]
8 premanifest HD patients, 8 manifest HD patients and 16 HC	^11^C-PK11195	In premanifest patients, significantly increased BP_ND_ radioligand in cognitive regions compared to normal controls Same results for symptomatic HD, with additional increases in pallidum, anterior prefrontal cortex and limbic striatum	Politis et al. (2011) [88]
12 premanifest HD patients vs. 12 HC	^11^C-PK11195	Significant increases in BP_ND_ radioligand in striatum, pallidum, thalamus and precentral gyrus in premanifest HD patients versus healthy controls	Politis et al. (2015) [89]

BP_ND_, binding potential; HD, Huntington’s disease; HC, healthy control.

**Table 4:** PET imaging studies assessing TSPO tracers in dementias.

Disorder	Population	Radioligand	Main Findings	References
**FTLD**	5 FTLD vs. 8 HC	^11^C-PK11195	Significantly increased radioligand binding in cortical frontal, medial temporal and subcortical areas in FTLD group	Cagnin et al. (2004) [130]
**FTDP-17**	3 presymptomatic MAPT gene carriers vs. 4 HC	^11^C-DAA1106	^11^C-DAA1106 BP_ND_ greater in the medial frontal, occipital and in the posterior cingulate cortex of pre-symptomatic gene carriers than in healthy volunteers	Miyoshi et al. (2010) [131]
**DLB**	11 early-stage drug-naive DLB patients vs. 11 HC	^11^C-PK11195	DLB patients showed increased subcortical (basal ganglia, substantia nigra) and cortical (cerebellum) tracer BP_ND_	Iannaccone et al. (2013) [54]
**PDD**	11 PDD patients vs. 10 HC	^11^C-PK11195	BP_ND_ significant increase in frontal, temporal, parietal and occipital cortical regions	Edison et al. (2013) [52]
**PDD/AD**	10 AD, 10 MCI, 11 PDD patients and 16 HC	^11^C-PK11195	Significantly increased tracer BP_ND_ in cortical and subcortical regions of AD, PDD and MCI patients compared with controls Significant negative correlation between MMSE and ^11^C-PK11195 BP_ND_ in temporoparietal, occipital and frontal cortex of AD and PDD patients	Fan et al. (2015) [125]
	9 PDD, 8 AD and 8 HC	^11^C-PK11195	Tracer was significantly increased in cortical regions of AD and PDD patients, compared to controls	Femminella et al. (2016) [132]
**AD**	8 AD patients vs. 15 HC	^11^C-PK11195	Significantly increased radioligand binding in the entorhinal, temporoparietal and cingulate cortex 1 patient with isolated memory impairment but without dementia exhibited an increase of ^11^C-PK11195 binding in the fusiform gyri, inferior temporal gyri and parahippocampus	Cagnin et al. (2001) [100]
	13 AD patients vs. 10 HC	^11^C-PK11195	Significantly increased tracer BP_ND_ in the cortical and subcortical regions in AD patients Tracer binding in gray matter and cortical regions negatively correlated with MMSE scores	Edison et al. (2008) [105]
	10 AD patients vs. 10 HC	^11^C-DAA1106	Significantly increased ^11^C-DAA1106 BP_ND_ in the cerebellum, cortical regions (prefrontal, lateral temporal, parietal and occipital cortex) and striatum in AD patients, compared to age-matched control subjects	Yasuno et al. (2008) [107]
	13 MCI patients vs. 10 HC	^11^C-PK11195	5/13 patients showed higher cortical tracer BP_ND_ than in controls Only frontal cortex radioligand uptake was significantly raised in MCI patients	Okello et al. (2009) [115]
	6 mild-moderate AD patients, 6 MCI patients and 5 HC	^11^C-PK11195	TSPO PET images showed no significant difference between the three groups	Wiley et al. (2009) [111]
	11 drug-naïve AD patients vs. 10 HC	^11^C-PK11195	Significantly increased ^11^C-PK11195 BP_ND_ in the medial frontal, parietal and left temporal cortex in the AD group Significant inverse correlation between MMSE score and ^11^C-PK11195 BP_ND_ in anterior cingulate cortex, precuneus and hippocampus	Yokokura et al. (2011) [106]
	6 AD patients vs. 12 HC	^11^C-vinpocetine	No significant difference between AD patients and age-matched control subjects in SUV of all brain regions	Gulyas et al. (2011) [133]
	10 AD, 7 MCI patients and 10 HC	^11^C-DAA1106	Mean BP values of MCI and AD patients higher than those of controls in prefrontal, parietal, temporal, occipital and cingulate cortex, striatum, cerebellum and thalamus No difference in binding was found between AD and MCI patients	Yasuno et al. (2012) [108]
	19 AD, 10 MCI patients and 13 HC	^11^C-PBR28	Significantly increased tracer binding in inferior parietal lobule, middle plus inferior temporal cortex and precuneus in AD patients, compared to MCI patients and HC EOAD patients had greater binding than LOAD	Kreisl et al. (2013) [109]
	19 probable AD, 10 MCI patients and 21 HC	^11^C-PK11195	ROI based analyses showed no differences between diagnostic groups, with large overlap between subject groups	Schuitemaker et al. (2013) [112]
	9 AD patients vs. 7 HC	^18^F-FEDAA1106	No significant change in ^18^F-FEDAA1106 binding between AD and HC	Varrone et al. (2013) [134]
	- 8 AD patients vs. 8 HC - AD patients were followed-up for 16 months	^11^C-PK11195	- AD patients showed a mean increase of 30% radioligand BP_ND_ in frontal, temporal, parietal, occipital cortical regions, hippocampus and striatum - In parallel to cognitive deterioration, microglial activation was increased longitudinally in 6/8 AD patients	Fan et al. (2015) [103]
	25 AD patients, 11 MCI and 21 HC	^11^C-PBR28	AD patients showed greater radioligand V_T_/f_P_ values than MCI patients and HC in combined middle, inferior temporal and entorhinal cortices	Lyoo et al. (2015) [102]
	10 AD patients vs. 7 HC	^18^F-FEMPA	Significantly higher radioligand V_T_ in the medial temporal cortex in AD patients than in controls	Varrone et al. (2015) [135]
	18 AD patients in moderate stage vs. 21 HC	^18^F-FEPPA	Compared to HC, AD patients showed a significant increase in ^18^F-FEPPA binding in both, gray matter (hippocampus, temporal, prefrontal, parietal and occipital cortex) and white matter (cingulum bundle, posterior limb of the internal capsule) No statistical correlation between radioligand V_T_ in white or gray matter and AD severity	Suridjan et al. (2015) [104]
	10 AD patients vs. 6 HC	^18^F-DPA-714	No significant difference in tracer BP_ND_ and V_T_ in whole brain gray matter between AD patients and HC	Golla et al. (2015) [136]
	- 14 AD patients vs. 8 HC - Follow-up of 2.5 years in AD patients	^11^C-PBR28	- Tracer binding (SUVr) significantly greater among patients than controls in inferior parietal lobule, precuneus, occipital cortex, hippocampus, entorhinal cortex and combined middle and inferior temporal cortex EOAD patients showed significantly higher TSPO radioligand binding than LOAD patients in several cortical regions (e.g., prefrontal and occipital cortex) - TSPO binding in temporoparietal areas increased from 3.9% to 6.3% per annum in patients, compared with only −0.5–1% per annum in controls	Kreisl et al. (2016) [101]
	- 58 AD patients (34 MCI and 24 dementia) vs. 32 controls (24 HC and 8 amyloidosis-controls) - 30 AD patients were followed-up over 2 years	^18^F-DPA-714	- In MAB + HAB group, tracer-GCI significantly higher in amyloidosis controls than in HC, especially in the cingulum and the precuneus Compared to controls, significantly increased tracer binding in temporal and parietal cortex in AD groups, even at the prodromal stage Significant positive correlation between the DPA-714-GCI and MMSE scores - GCI-DPA-714 SUVr greater in the slow decliners than in the fast decliners group	Hamelin et al. (2016) [110]

AD, Alzheimer’s disease; BP_ND_, non-displaceable binding potential; CDR-SB, clinical dementia rating scale-sum of boxes; DLB, dementia with Lewy bodies; EOAD, early-onset AD; FTDP-17, frontotemporal dementia with parkinsonism linked to chromosome 17; FTLD, frontotemporal lobar degeneration; f_P_, plasma-free fraction of radioligand; GCI, global cortical index; HAB, TSPO high affinity binders; HC, healthy control; LOAD, late-onset AD; MAB, TSPO mixed affinity binders, MCI, mild cognitive impairment; MMSE, mini-mental state examination; ROI, region of interest, SUVr, standard uptake value ratio; PPD, Parkinson’s disease dementia; V_T_, total distribution volume.

**Table 5:** PET imaging studies assessing TSPO tracers in amyotrophic lateral sclerosis.

Population	Radioligand	Main Findings	References
10 ALS patients without dementia (2 bulbar-onset and 8 limb-onset) – mean disease duration = 25 months	^11^C-PK11195	The difference in tracer binding between ALS and controls reached significance in motor cortex, pons, frontal lobe region and thalamus Statistical correlation between BP_ND_ values and UMNB scores for both motor cortex and thalamus was found No clear difference in the binding of ^11^C-PK11195 between ALS patients with bulbar- or limb-onset No correlation between tracer uptake with either disease duration or ALSFRS-R was seen	Turner et al. (2004) [32]
10 riluzole-naive ALS patients without dementia (6 bulbar-onset and 4 limb-onset) vs. 8 HC – mean disease duration = 12 months	^18^F-DPA-714	Primary motor, supplementary motor and temporal regions ^18^F-DPA-714 DVr values were greater in ALS patients than in age-matched healthy volunteers No significant difference in occipital and frontal cortex, thalamus, cerebellum or pons was found between the two groups No correlation between DVr values and disease duration or ALSFRS-R	Corcia et al. (2012) [148]
10 ALS patients (3 bulbar-onset and 7 limb-onset) vs. 10 HC – mean disease duration = 22 months	^11^C-PBR28	Significantly increased tracer binding in the primary motor cortex and upper region of the corticospinal tract in ALS patients Tracer SUVr of the right precentral gyrus was positively correlated with UMNB scores and negatively correlated with ALSFRS-R No statistical correlation was found between radioligand uptake in the precentral gyrus and disease duration ALS patients with limb-onset showed significantly higher SUVr than patients with bulbar onset in the precentral gyrus	Zürcher et al. (2015) [147]

ALS, amyotrophic lateral sclerosis; ALSFRS-R, amyotrophic lateral sclerosis functional rating scale revised; BP_ND_, binding potential; DVr, distribution volume ratio; HC, healthy control, SUVr, standard uptake value ratio; UMNB, upper motor neuron burden scale.

**Table 6:** PET imaging studies assessing TSPO tracers in psychiatric disorders.

Disorder	Population	Radioligand	Main Findings	References
BD	14 patients with BD-I vs. 11 HC	^11^C-PK11195	No significant difference in the whole-brain gray matter BP in patients with bipolar I disorder versus healthy subjects Significantly increased tracer binding in hippocampus of BD-I patients, compared to normal controls	Haarman et al. (2014, 2016) [155,170]
MDE	10 patients with mild to moderate depression vs. 10 HC	^11^C-PBR28	No statistically significant difference in ^11^C-PBR28 V_T_ between individuals with depression and healthy volunteers 7/10 patients exhibited lower tracer V_T_ in cortical regions (frontal, temporal, parietal and occipital), striatum, thalamus and cerebellum than normal controls	Hannestad et al. (2013) [167]
	20 MDE drug-free patients vs. 20 HC	^18^F-FEPPA	In MDE patients, significant elevation of TSPO V_T_ in the prefrontal cortex, anterior cingulate cortex and insula, of 26%, 32% and 33%, respectively, compared to normal controls Anterior cingulate cortex TSPO V_T_ correlated with total 17-item HDRS score	Setiawan et al. (2015) [168]
SCZ	10 patients with recent-onset SCZ vs. 10 HC	^11^C-PK11195	Significantly greater total gray matter tracer BP in SCZ patients than in healthy volunteers No statistical correlation between total gray matter ^11^C-PK11195 BP and PANSS	Van Berckel et al. (2008) [171]
	7 SCZ patients vs. 8 HC	^11^C-PK11195	Significantly higher radioligand BP was observed in the hippocampus of SCZ patients than in HC Although the difference did not reach a significance level, the whole-brain gray matter and white matter were, respectively, 30% higher and 20% greater in SCZ patients than in HC	Doorduin et al. (2009) [172]
	14 SCZ patients vs. 14 HC	^11^C-DAA1106	No clear difference in either regional or total cortical radioligand BP_ND_ values between the two groups	Takano et al. (2010) [173]
	16 SCZ patients vs. 27 HC	^18^F-FEPPA	No significant effect of clinical group (SCZ vs. HC) detected on tracer V_T_ in hippocampus, medial prefrontal cortex, dorsolateral prefrontal cortex, temporal cortex and striatum or in white matter tracts None of the disease parameters (PANSS, AES and RBANS) statistically correlated with regional radioligand V_T_	Kenk et al. (2015) [174]
	14 UHRP subjects (APT-naive), 14 SCZ patients and 28 HC	^11^C-PBR28	Significantly increased tracer DVr in total gray matter and frontal and temporal lobes in UHRP subjects and SCZ patients, compared to age-matched controls No statistical difference in tracer binding values between UHRP and SCZ patients In UHRP subjects, statistical positive correlation was found between CAARMS and ^11^C-PBR28 DVr in total gray matter	Bloomfield et al. (2016) [175]
	12 recent onset SCZ patients vs. 14 HC	^11^C-DPA713	Significantly increased ^11^C-DPA713 V_T_ in amygdala and cingulate cortex in SCZ patients compared healthy volunteers	Coughlin et al. (2016) [176]
	12 recent onset SCZ patients vs. 14 HC	^11^C-DPA713	Relative to HC, there was a strong trend towards reduce ^11^C-DPA713 V_T_ in the middle frontal gyrus of patients with recent onset SCZ	Notter et al. (2017) [177]

AES, Apathy Evaluation Scale; APT, antipsychotic therapy; BD, bipolar disorder; BP, binding potential; BP_ND_, binding potential; CAARMS, comprehensive assessment of the at-risk mental states; CDS, calgary depression scale; DVr, distribution volume ratio; IDC-C_30_, inventory of depressive symptoms—clinician version; HDRS, Hamilton depression rating scale; HC, healthy control; MDE, major depressive episodes; PANSS, positive and negative syndrome scale; RBANS, repeatable battery for the assessment of neuropsychological status; SCZ, schizophrenia; UHRP, ultra-high risk of psychosis; V_T_, total distribution volume.

**Table 7:** PET imaging studies assessing TSPO tracers in multiple sclerosis.

Population	Radioligand	Main Findings	References
2 RR patients	^11^C-PK11195	Increased ^11^C-PK11195 BP_ND_ in MRI-defined active MS lesions, not in chronic lesions	Vowinckel et al. (1997) [213]
12 MS patients (8 RR, 1 SP and 3 PP), 8 HC	^11^C-PK11195	Increased global and in MRI-defined (T_1_/T_2_) active lesions radioligand BP_ND_ in MS patients Greater brainstem and thalamus tracer BP_ND_ in MS patients than in HC Patient with a history of optic neuritis showed ^11^C-PK11195 signals in the lateral geniculate bodies (to which the optic nerve projects)	Banati et al. (2000) [210]
22 MS patients (13 RR, 7 SP and 2 PP), 7 HC	^11^C-PK11195	No statistical difference in NAWM and GM tracer BP_ND_ was found between MS patients and healthy volunteers Tracer BP_ND_ significantly greater in gadolinium lesions than in NAWM A higher ^11^C-PK11195 BP_ND_ was found in both situations, clinical relapse or presence of T1-gadolinium lesions Statistical correlation found between higher ^11^C-PK11195 BP_ND_ and disease duration	Debruyne et al. (2003) [199]
22 MS patients (13 RR, 7 SP and 2 PP), 8 HC	^11^C-PK11195	NAWM tracer BP_ND_ was positively correlated with amount of brain atrophy Significant negative correlation between T_2_ lesions tracer BP_ND_ and brain atrophy	Versijpt et al. (2005) [214]
11 MS (mostly RR) patients vs. 7 HC	^11^C-PBR28	No statistical difference in neither WM fraction or GM fraction V_T_ tracer between MS and healthy volunteers Gadolinium-enhancing lesions demonstrated significantly higher ^11^C-PBR28 binding than contralateral NAWM Global V_T_ tracer showed correlation with disease duration but not with clinical disability	Oh et al. (2011) [203]
18 MS patients (10 RR and 8 SP), 8 HC	^11^C-PK11195	Proportion of abnormal high total cortical GM ^11^C-PK11195 BP_ND_ was greater in SP than in RR patients Mean total WM radioligand BP_ND_ was greater in SP than in RR patients SP patients showed additionally increased tracer BP_ND_ in precentral, superior parietal, lingual and anterior superior, median and inferior temporal gyri The 2 patients with the more active RR had the highest total cortical GM tracer BP_ND_	Politis et al. (2012) [198]
9 RR untreated patients Patients were rescanned after 1 year of GA	^11^C-PK11195	The patient with the more active RR had the highest ^11^C-PK11195 BP_ND_ on the baseline TSPO PET scan Tracer BP_ND_ per unit volume statistically decreased in the whole brain and especially in the following regions: supratentorial brain, infratentorial brain, cerebral white matter, cortical gray matter, thalamus and putamen	Ratchford et al. (2012) [204]
9 RR in acute relapse (6 without treatment and 3 interferon-β treated) vs. 5 HC	^18^F-FEDAA1106	Similar radioligand BP_ND_ and V_T_ between normal controls and MS patients No significant difference of BP_ND_ or V_T_ between MS patients without treatment, MS patients with interferon-β treatment and healthy volunteers	Takano et al. (2013) [215]
11 RR vs. 11 HC	^18^F-PBR111	Increased tracer uptake in WM lesions and perilesional WM relative to HC Increased ^18^F-PBR111 V_T_ was higher in lesions and perilesional volumes in MS patients compared to the NAWM of the same subjects Significant positive correlation between radioligand V_T_ and MS score severity	Colasanti et al. (2014) [205]
19 MS patients (10 RR, 8 SP and 1 PP)	^11^C-PK11195	Black holes tracer BP_ND_ was greater in progressive patients than in RR patients In RR patients, significant inverse correlation between volume and ^11^C-PK11195 BP_ND_ of black holes was found Black holes tracer BP_ND_ was correlated with the EDSS in progressive patients	Giannetti et al. (2014) [206]
10 SP patients vs. 8 HC	^11^C-PK11195	Relative to controls, significantly increased tracer DVR was found in periventricular NAWM and thalamus of SP patients A pattern of increased TSPO density in the periplaque area surrounding the borders of chronic plaques was observed In active plaques with gadolinium-enhancement increased TSPO binding was found in the core of the lesion but not in the area surrounding them	Rissanen et al. (2014) [202]
18 CIS patients vs. 8 HC	^11^C-PK11195	Compared to controls, significantly increased tracer BP_ND_ was found in NAWM of CIS patients, while no difference was observed in GM In CIS patients with T_2_ MRI lesions, NAWM tracer BP_ND_ correlated with EDSS CIS patients who developed MS at 2 years had higher ^11^C-PK11195 BP_ND_ at baseline	Giannetti et al. (2015) [206]
5 RR patients (4 stable and 1 drug naive with active RR) vs. 4 HC	^11^C-PBR28	V_T_ was similar between stable MS and healthy volunteers across whole brain and GM/WM/NAWM regional areas (frontal, parietal, temporal and occipital) Focal increases in tracer V_T_ were observed at some areas corresponding to gadolinium-enhancing lesion of active MS patients	Park et al. (2015) [216]
9 RR patients vs. 8 HC	^18^F-GE180	Whole WM uptake (SUVR_GM_) slightly higher in RR patients than in HC Increased tracer uptake in inactive WM lesions and a further increase in gadolinium-enhancing lesions (Preliminary data)	Trigg et al. (2015) [217]
11 RR patients (6 with MDE) vs. 22 HC	^18^F-PBR111	^18^F-PBR111 DVR higher in the hippocampus of MS patients relative to normal subjects, while no difference was observed in thalamus Hippocampal radioligand binding was correlated to BDI scores in MS patients and was highest in MS patients with current MDE	Colasanti et al. (2016) [218]
23 MS patients (16 RR and 7 SP)	^11^C-PBR28	No significant correlation between ^11^C-PBR28 DVR and EDSS in either WM lesions, NAWN or GM	Datta et al. (2016) [211]
27 MS patients (12 RR and 15 SP), 14 HC	^11^C-PBR28	Compared to controls, significantly increased tracer DVR across the brain was found in MS cases; the greatest increases being in cortex and cortical lesions, thalamus, hippocampus and NAWM Relative to the same brain regions, radioligand uptake was greater in SPMS than in RRMS, except for cortical lesions (same TSPO levels) In MS patients, EDSS correlated positively with increased tracer SUVR in the NAWM, WM lesions, thalamus, hippocampus and basal ganglia	Herranz et al. (2016) [200]

BP_ND_, binding potential; BDI, beck depression inventory; CIS, clinically isolated syndrome; DVR, distribution volume ratio; EDSS, expanded disability status scale; GA, glatiramer acetate; GM, gray matter; HC, healthy control; MDE, major depressive disorder; MS, multiple sclerosis; NAGM, normal-appearing gray matter; NAWM, normal-appearing white matter; PP, primary progressive multiple sclerosis; RR, relapse-remitting multiple sclerosis; SP, secondary progressive multiple sclerosis; SUVR, standardized uptake value ratio; V_T_, total volume of distribution; WM, white matter.
